# Potential of Urolithin A to improve joint health

**DOI:** 10.18632/aging.204633

**Published:** 2023-03-28

**Authors:** Davide D’Amico, Martin Lotz

**Affiliations:** 1 Amazentis SA, EPFL Innovation Park, Lausanne, Switzerland; 2 Department of Molecular Medicine, Scripps Research, La Jolla, CA 92037, USA

**Keywords:** osteoarthritis, Urolithin A, mitochondria, mitophagy, inflammation

There is growing interest in improving the quality of life as we get older. The ability to move freely is key for an increased health span. And the health of our joints plays a pivotal role in that. However, joint function tends to decline with age, overuse, or in certain genetic diseases. This happens when cartilage and other tissues within the joint start to deteriorate, and can lead to the most common form of arthritis, osteoarthritis (OA) [1]. Compromised joint function and impaired overall mobility are closely linked with joint pain, the lead symptom of patients with OA [1].

The condition is already a leading cause of work disability in the United States and a major economic burden in an increasingly older human population. There is no effective therapy and for many OA patients the best they can expect is pain management which is also in need for more effective and safer medications for chronic use.

Our recent work demonstrates a new and promising approach. Urolithin A, a natural postbiotic molecule showed significant protection against OA associated disease markers [2]. This effect was associated with an increased quantity and quality of mitochondria in joint cartilage cells, the chondrocytes [2].

Mitochondrial dysfunction is a hallmark of aging [3]. This derives from reduced production of functional organelles and an accumulation of faulty mitochondria. The consequences are insufficient energy production to support cell functions and a buildup of toxic byproducts that are detrimental for cellular health. A promising strategy to reverse mitochondrial dysfunction is to enhance mitophagy, the cellular process to remove and recycle damaged mitochondria [4]. This clears the cells of dysfunctional organelles and, in turn, instructs cells to regenerate healthy organelles.

Urolithin A has been shown to improve mitophagy and mitochondrial function in several tissues affected by aging and age-associated diseases, particularly skeletal muscle [5]. Preclinically, UA enhanced mitochondrial quality in rodent models of aging and muscle dystrophy [5]. In independent, double-blind placebo-controlled human clinical studies, this safe and natural molecule both improved muscle strength in middle-aged adults [6] and muscle endurance in healthy elderly people [7]. Our recent study published in Aging Cell, extends the benefits of UA to the joint [2].

The data showed human primary chondrocytes treated with UA had increased mitophagy flux and higher levels of PINK1-Parkin mediated mitophagy. This increased mitophagy was associated with enhanced mitochondrial respiration, indicative of higher energy production in cartilage cells. An increase in mitophagy and mitochondrial abundance in joint tissue was also shown *in vivo*, in a preclinical model of knee OA where UA was administered UA for two months. Importantly this was accompanied by significant improvement in the cartilage tissue quality. The untreated controls exhibited standard features of OA joints, such as cartilage erosion and increased cell death. Supplementation with UA improved these pathological aspects, leading to better quality and cellularity of the knee joints.

Another common hallmark of aging is low-grade, chronic inflammation, also known as inflamm-aging. And inflammation is one of the main mechanisms of joint tissue damage and OA-associated pain. There is extensive literature showing UA to have anti-inflammatory activity in several preclinical models [5].

This effect has been translated also in humans. A 4 months administration of UA led to reduced circulating levels of CRP, a well-established marker of inflammation, in subjects that are not diseased but have suboptimal health status [6, 7]. Notably, a mild anti-inflammatory effect of UA was also shown in the OA models described above, with reduced synovium inflammation (synovitis) and lower levels of the circulating OA-promoting biomarker MMP1 [2].

Taken together, the study of the impact of UA in OA models indicates that UA’s beneficial effects could derive from a combined effect on two aging-relevant physiological changes: improved mitochondrial quality and reduced inflammation. Further mechanistic studies should reveal the exact interplay between UA and changes on these two biological pathways.

Another key observation is that UA increases mitophagy and mitochondrial not only in cells from OA patients, but also in chondrocytes from healthy subjects. Further *in vivo* studies are required, but these initial data suggest UA may support joint health even before the onset of disease.

**Figure 1:**
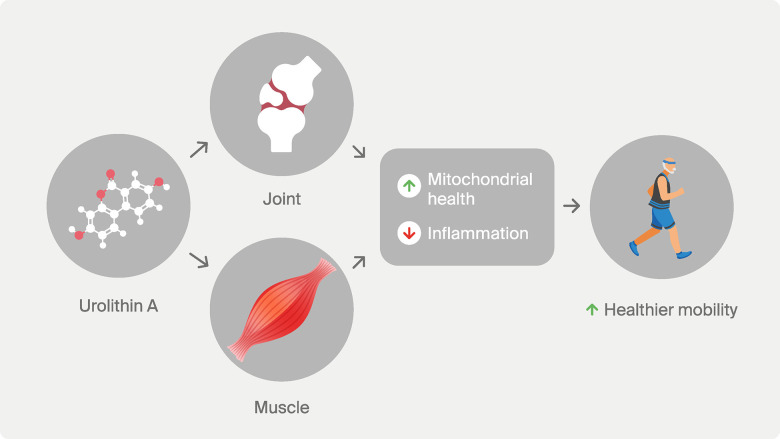
**Impact of Urolithin A on healthy mobility.** Several preclinical and clinical works indicate Urolithin A’s potential to improve mobility through a combined effect on joint and muscle tissues. Mechanistically, the beneficial effect on Urolithin A on healthy mobility is associated with increased biomarkers of mitochondrial health and reduced biomarkers of inflammation.

Finally, building on the existing literature on Urolithin A, the Aging Cell study supports a combined role for UA in preserving healthy mobility, by acting on both cartilage cells and skeletal muscle (Figure 1). Other reports also show additional beneficial effects of UA for bone repair [8]. A fascinating question is whether the biological effects of UA in different musculoskeletal organs are linked by inter-tissue crosstalk. Certainly, multiple clinical studies show improved muscle health with UA. Additional trials could now investigate the benefits of UA in joint health and other applications to support healthy aging.

In summary, UA’s impact on mitochondrial function provides benefits to multiple tissues and their corresponding physiological functions. This suggests regular supplementation of UA may be a useful way to help counter a number of functional and biological changes that occur with aging. This latest data further supports the potential of UA as a nutritional intervention to support joint function and to help people retain better movement and mobility as they grow older.
